# Catalytic activity of catalase–silica nanoparticle hybrids: from ensemble to individual entity activity†
†Electronic supplementary information (ESI) available. See DOI: 10.1039/c6sc04921d



**DOI:** 10.1039/c6sc04921d

**Published:** 2016-12-15

**Authors:** Crystal Chan, Lior Sepunaru, Stanislav V. Sokolov, Enno Kätelhön, Neil P. Young, Richard G. Compton

**Affiliations:** a Department of Chemistry , Physical & Theoretical Chemistry Laboratory , University of Oxford , South Parks Road , Oxford OX1 3QZ , UK . Email: richard.compton@chem.ox.ac.uk; b Department of Materials , University of Oxford , OX1 3PH , UK

## Abstract

We demonstrate the electrochemical detection and characterization of individual nanoparticle–enzyme hybrids. Silica nanoparticles were functionalized with catalase enzyme and investigated spectroscopically and electrochemically. The catalytic activity of the hybrids towards hydrogen peroxide decomposition was comparable to the activity of a freely diffusing enzyme in solution, exhibiting a Michaelis–Menten constant of *K*_M_ = 74 mM and a turnover number of *k*_cat_ = 8 × 10^7^ s^–1^ per NP. The fast turnover number of the hybrid further enabled the electrochemical detection of individual nanoparticle–enzyme hybrid *via* a novel method: the hydrogen peroxide substrate was generated at a microelectrode which enabled enzymatic activity exclusively within the diffusion layer of the electrode. The method is the first electrochemical approach for measuring hybrid nanoparticles, at the single entity level.

## Abstract 2

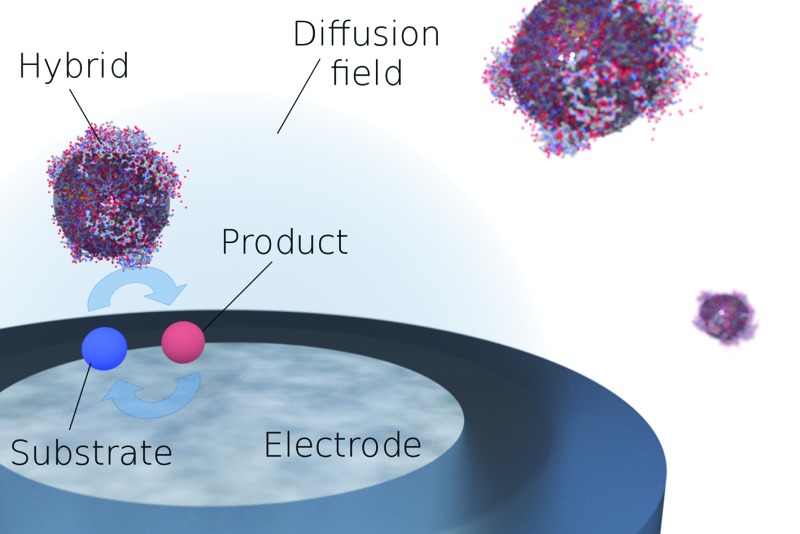
We demonstrate the electrochemical detection and characterization of individual nanoparticle–enzyme hybrids.

## Introduction

The incorporation of proteins into nanoparticle hybrids is relativity new and a novel approach to catalysis.^1–3^ The design of such hybrids enables integrating selective catalysis and recognition elements in a confined space defined by the nanoparticle size. Enzyme/NP hybrids can be used for a broad range of applications such as the synthesis of new biocatalysts or drugs,^4,5^ biosensing assays,^6^ biomedical^7^ and nanobioelectronic devices.^8,9^ The preparation of enzyme/NP hybrids can lead to various changes such as reduced enzymatic activity^10^ or partial denaturation,^11^ together with agglomeration of the as synthesized bare NP.^12^ Lately, silica nanoparticles (SiNP) have been recognized as a suitable platform for biological modification, as the properties of the bio-component and the NP may remain unaltered.^13^ Recently and ‘unrelated at first sight’, a new electrochemical method has emerged^14–16^ aiming to unravel electrochemical activity at the single entity level. This electrochemical technique provides the opportunity to characterize single nanoparticles^17–19^ *in situ* and large macromolecules in solution.^20–23^ Herein, we further extend the applicability of the electrochemical method at a single entity level, and demonstrate that the activity of a single NP/enzyme hybrid can be detected and quantified.

## Results and discussion

**Fig. 1:**
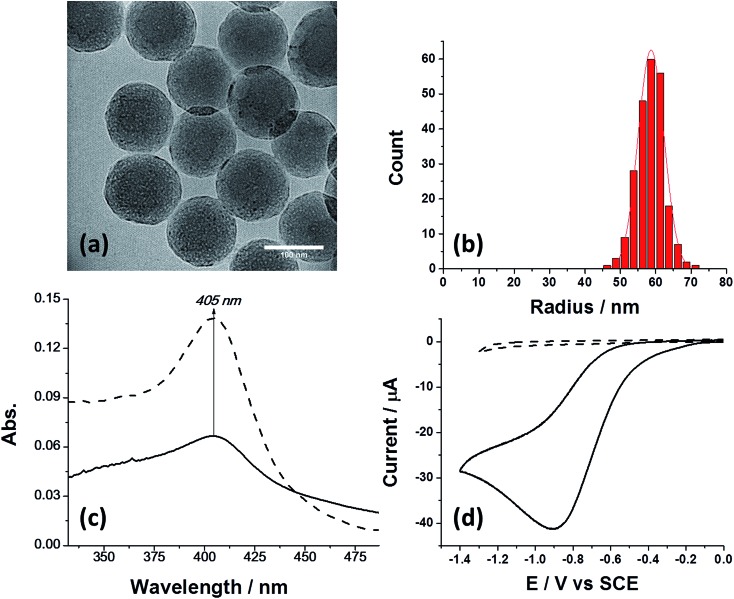
Characterization of the SiNPs covered with catalase. (a) TEM image of the SiNPs functionalized with catalase. (b) Statistical distribution of the NP size as extracted from TEM images of over 230 NPs. (c) UV-Vis absorption of 0.4 μM catalase freely-diffusing in solution (dashed) and when immobilized on 0.5 nM SiNPs in solution (solid). (d) Cyclic voltammetry of drop cast SiNPs with (solid) and without (dashed) immobilized catalase. 4 μL of 300 pM SiNPs (pH 5.4, with and without catalase modification) were drop cast on a GCE electrode and exposed to a 2 mM H_2_O_2_ solution depleted of oxygen. Temperature was 25 °C and scan rate was 50 mV s^–1^.

The preparation of SiNPs (nanoComposix, CA, USA) modified with bovine catalase was done according to a previously reported protocol^24^ with slight modifications (see ESI†). First, the nanoparticles with and without catalase modification were characterized by TEM (Fig. 1a), nanoparticle tracking analysis (NTA), and zeta potential measurements (Fig. S1a†). The mean size and standard deviation of the SiNPs was *r* = 58.6 ± 3.7 nm (Fig. 1b), which is in excellent agreement with the manufacturer specification for the bare SiNPs (*r* = 59.2 ± 2.8 nm). The size of the modified SiNP was unaltered upon catalase modification, as seen from the TEM images of the unmodified SiNPs (Fig. S1b†). Next, we quantified the average amount of enzymes immobilized on the nanoparticle. Fig. 1c shows the absorption spectra of a solution containing 0.4 μM catalase (dashed line) and a solution containing 0.5 nM hybrid particles (solid line). It is evident that the absorption peak at 405 nm, which corresponds to the catalytic iron centre^25^ was conserved after enzyme immobilization on the SiNPs. The bare SiNPs without catalase had no absorption maxima (Fig. S2†). Since the number of nanoparticles in solution was determined from the NTA and the enzyme extinction coefficient is known to be (*ε* = 340 000 M^–1^ cm^–1^ (ref. 26)), we were able to estimate the average number of enzymes immobilized on a SiNP. The extracted number is close to that expected for monolayer formation and corresponds to an average of 370 ± 24 catalase enzyme immobilized per SiNP (see calculation in ESI†). As will be shown later, the SiNP size of ∼60 nm was chosen as a suitable platform for a sufficient number of immobilized enzymes which consequently produced a detectable electrochemical signal. Next, we investigated the electrochemical properties of the SiNP/catalase hybrids. For electrochemical experiments a glassy carbon macro-electrode (GCE, *r* = 1.5 mm) was used as a working electrode together with a saturated calomel electrode (SCE) and a Pt wire serving as the reference- and counter electrode, respectively. First we verified that the bare SiNPs were not electrochemically active towards hydrogen peroxide. As shown in Fig. 1d (dashed line), a featureless voltammogram was observed when unmodified SiNPs were drop cast on the surface (4 μL of 300 pM solution) in an oxygen free solution containing 2 mM of H_2_O_2_. The results indicate that the SiNPs are inert towards hydrogen peroxide in the potential range studied.

**Fig. 2:**
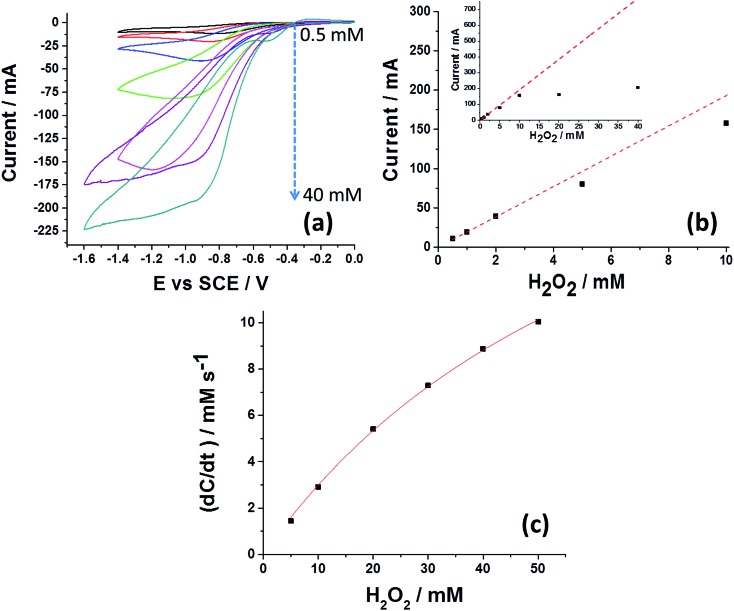
Activity of SiNP hybrid on an electrode surface and in solution. (a) Voltammetry of the SiNP/catalase hybrid drop cast on a GCE electrode. 4 μL of 300 pM SiNP/Cat were used in an oxygen free solution with 0.5, 1, 2, 5, 10, 20 and 40 mM H_2_O_2_ (pH = 5.4). Scan rate in all experiments was 50 mV s^–1^ with temperature of 25 °C. (b) Peak current as a function of H_2_O_2_ concentration. The dashed line reflects the theoretical diffusion-limited irreversible two electron reduction and the solid squares show the experimentally measured currents. (c) Reaction rate of the hydrogen peroxide disproportionation by 0.3 pM SiNP/Cat in a solution of pH = 5.4 using UV-Vis spectroscopy at 240 nm. The extinction coefficient used for H_2_O_2_ was (*ε* = 43.6 M^–1^ cm^–1^).^33^

However, when the same experimental conditions were used for drop cast SiNPs modified with catalase, a clear reduction peak is observed (Fig. 1d, solid line). In a control experiment, a solution that was bubbled with oxygen, *i.e.*, a ‘saturated oxygen solution’ without hydrogen peroxide, produced a voltammogram with similar reduction potential that is consistent with oxygen reduction on a carbon electrode (Fig. S3†).^27^ Closer examination of the oxygen reduction process on a bare GCE and a SiNP modified GCE reveals that the reduction occurs at higher overpotential for the latter, consistent with an additional resistive layer, such as SiNPs. Further reduction of hydrogen peroxide to water on a GCE occurs at a higher reductive potential (∼–1.3 V *vs.* SCE), outside the potential range used (Fig. S3†). Therefore, it can be concluded that the SiNP hybrids on the GCE decompose the hydrogen peroxide into oxygen which is further reduced electrochemically on the GCE. The catalytic activity of the SiNP/catalase ensemble was further investigated spectroscopically and electrochemically. In order to elucidate the mechanism governing the observed reduction, the GCE modified with the SiNP/catalase hybrid was exposed to various concentrations of hydrogen peroxide in an oxygen free solution. A clear increase in the peak current of the oxygen reduction as a function of the hydrogen peroxide concentration is seen in Fig. 2a. This data is consistent with the modified electrode operating in a diffusion-limited process where the peak current reflects the depletion of hydrogen peroxide at the electrode surface during the voltammetric scan.^28^ At hydrogen peroxide concentrations higher than 10 mM the peak current is no longer observed and a transformation to a ‘quasi’ steady state current appears. Under this condition, the mass transport of hydrogen peroxide to the electrode is no longer the dominant mechanism that controls the current magnitude and the enzyme kinetics become the rate limiting step.^29^ It is worth noting, that in this case, the enzymatic product O_2_ is detected and a direct charge transfer from the enzyme to the electrode is absent. The following mechanism describes the above CE reaction:^28^

1 SiNP/Cat + 2H_2_O_2_ → SiNP/Cat + O_2_ + 2H_2_O

2 O_2_ + 2H^+^ + 2e^–^ → H_2_O_2_ at the electrode

In the scheme, hydrogen peroxide is first decomposed by the SiNP/catalase hybrid to produce oxygen and water (eqn (1)). Next, the enzymatically produced oxygen is electrochemically reduced *via* a two electron process at the GCE^27^ which regenerates hydrogen peroxide (eqn (2)). The regenerated hydrogen peroxide can be further decomposed by the SiNP/catalase hybrids. Theoretically, in an ideal cycle, a total of up to two electrons can be produced from one hydrogen peroxide molecule at low over-potentials. Fig. 2b shows the experimentally observed peak current as a function of the hydrogen peroxide concentration together with the theoretical value (see further explanation in the ESI†) for a diffusion-limited irreversible two-electron reduction of hydrogen peroxide on a glassy carbon macro-electrode. At low concentrations of hydrogen peroxide the peak current approximates a full two electron reduction. However, as the concentration of hydrogen peroxide is increased, the flux of hydrogen peroxide to and from the electrode becomes more rapid. Consequently, at sufficiently high rates of mass transport the enzyme kinetics dominate the total reaction rate (Fig. 2b inset). From the obtained voltammograms it is clear that the catalase enzyme immobilized on the SiNP are active. We have further investigated the efficiency of the catalytic process *via* UV-Vis spectroscopy. This was done by measuring the rate of hydrogen peroxide decomposition (at 240 nm (ref. 30)) at a fixed concentration of 0.3 pM SiNP/Cat and various concentrations of hydrogen peroxide. Fig. 2c presents the measured reaction rate in a solution of 5–50 mM H_2_O_2_ (for the full UV-Vis kinetics please see Fig. S4†). The Michaelis–Menten constant *K*_M_ and the turnover number *k*_cat_ were determined^31^ *via* a nonlinear fit (Origin, hyperbl function, solid line) and values of *K*_M_ = 74 mM and *k*_cat_ = 8.3 × 10^7^ s^–1^ per NP were extracted. Since on average about 370 enzymes cover the SiNP surface, the catalytic turnover number per enzyme can be approximated to *k*_cat_ = 2.2 × 10^5^ s^–1^ (see Fig. S5† for reciprocal Lineweaver–Burk plot). Both *K*_M_ and *k*_cat_ are in good agreement with previously reported literature values.^32^

**Fig. 3:**
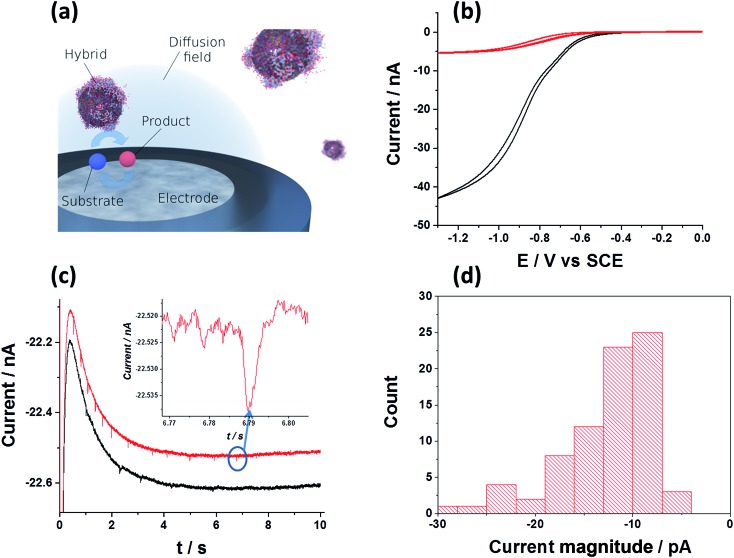
(a) Schematic of the characterisation of individual hybrid: surface induced catalysis within the diffusion layer and product detection at a microelectrode. (b) Oxygen reduction voltammograms of ‘saturated oxygen solution’ (upper curve, [O_2_] = 1.24 mM (ref. 37)) and of ‘super saturated’ oxygen solution induced by high concentration of 100 pM SiNP/catalase and 20 mM of H_2_O_2_. Measurements were done in citric-phosphate buffer solution (pH = 5.4) at 10 mV s^–1^ scan rate at 25 °C. (c) Examples of two chronoamperometric curves. The carbon microelectrode was held at a potential of –1.4 V *vs.* SCE to assure sufficient reductive potential. (Inset) typical current spike seen. (d) Statistical distribution of the current spike magnitude.

Hitherto, the electrochemical properties of NP/enzyme hybrids were solely probed in an ensemble.^8,34,35^ We can, however, exploit the fast decomposition rate of one hydrogen peroxide molecule per 10 to 20 ns and per hybrid to detect the presence of individual hybrids at the electrode. While in a previous study it was shown that the detection of individual enzymes is challenging and may even be impossible, if the substrate concentration is homogeneous and the experimental system resembles a one-dimensional diffusion scenario,^36^ we here pursue a different approach: in the presented system, the hydrogen peroxide substrate is only present in a confined space adjacent to the electrode surface, which is achieved *via* the generation of the substrate at the microelectrode. Since the diffusive mass transport towards and away from a microelectrode exhibits radial rather than linear diffusion characteristics, the substrate is exclusively present in the diffusion layer of the electrode. Enzymatic activity is hence suppressed in the bulk, where substrate is absent, and solely enabled in the proximity of the electrode, where individual hybrids can be detected and characterised (Fig. 3a). In oxygen containing solution, freely diffusing SiNP/Cat hybrid will not perform catalytic activity unless located in close proximity to the electrode, where hydrogen peroxide is generated. Further to this, the realisation of surface induced hydrogen peroxide formation on a carbon microelectrode, is shown in Fig. 3b.

Here, the GCE was replaced with a carbon micro-electrode (*r* = 3.5 μm) in order to achieve a high signal-to-noise ratio. Under constant bubbling of the solution with oxygen, a ‘saturated oxygen solution’ can be formed, with up to 1.24 mM of oxygen (Fig. 3b upper curve).^37^ However, a ‘super saturated’ oxygen solution, exceeding the latter concentration can be achieved using a high concentration of SiNP/Cat (100 pM) and hydrogen peroxide (10 mM) in solution. The process is conceptually similar to the formation of a ‘super saturated’ oxygen solution in sea water due for example to biological activity of photosynthetic species.^38^ As can be seen in Fig. 3b (lower curve), under these conditions, most of the hydrogen peroxide is converted rapidly (within seconds) into oxygen due to the high concentration of the NP/catalase hybrid (100 pM of SiNP ∼3.2 nM Cat). The half wave reduction potential seen on the microelectrode is a typical oxygen reduction signal (see comparison of the normalized currents from both voltammograms in Fig. S6†). Next, we show a current–time measurement at a fixed applied potential (–1.4 V *vs.* SCE) from a ‘super saturated’ oxygen solution that contains 100 pM SiNP/catalase and 20 mM hydrogen peroxide rapidly decomposed to ∼10 mM oxygen (as estimated from the observed steady state current). Here, we were able to produce high concentration of hydrogen peroxide at the electrode surface. As can be seen from Fig. 3c, current spikes are noticeable under applied potential of –1.4 V *vs.* SCE. No current spikes were seen in a 20 mM H_2_O_2_ solution with 100 pM of unmodified SiNP (Fig. S7†).

We attribute these currents to a process of random single NP hybrid operating exclusively within close proximity to the electrode due to Brownian motion of the particles in solution. Upon close proximity of an individual NP hybrid to the micro-electrode, the surface induced hydrogen peroxide is decomposed back to oxygen by the SiNP/catalase hybrid. Consequently, a transient oxygen reduction signal is observed. The frequency of the observed spikes was lower than the theoretically expected value for random impacts of the particles at an unshielded microelectrode (see ESI†). This is explained by an irreversible absorption process of the NP/catalase hybrids to the insulating glass surrounding the active microelectrode.^39^ The amplitude of each current spike is directly proportional to the maximum rate of oxygen production by a single hybrid. Statistical analysis of the current spikes produced a mean current around 10 pA (Fig. 3d). The current can be expressed in *k*_cat_ units (s^–1^) using the following relation:3
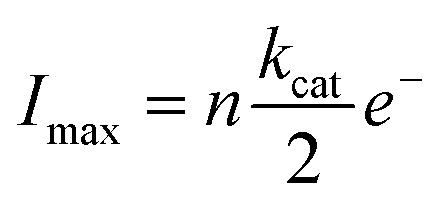
where *n* = 2 is the number of electrons transferred during the oxygen reduction process,^27^ 
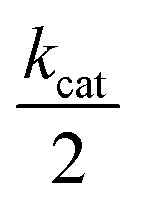
 reflects the rate of oxygen formation by the NP (s^–1^) and *e* ^–^ is the elementary charge (1.6 × 10^–19^ C). Using the value of *k*_cat_ = 8.3 × 10^7^ s^–1^ per NP hybrid calculated from the absorption kinetics, an excellent correlation between the theoretical maximum current and the experimentally observed current magnitude is achieved. Each NP hybrid produces oxygen molecules *via* the enzymatic route only when in close proximity to the electrode (*i.e.*, within the diffusion layer) which are subsequently fully consumed at the microelectrode.

## Conclusions

We demonstrated that a SiNP can be functionalized with catalase enzyme at a high surface coverage. The NP/catalase hybrids are stable, without noticeable aggregation. In addition, the catalytic activity of enzymes immobilized on SiNPs surfaces is conserved as shown electrochemically and by UV-Vis spectroscopy. On average each SiNP was covered with ∼370 enzymes. Hence, the current generated by a single NP at a microelectrode, due to oxygen product formation was sufficiently high to be observed using state of the art electronic components.^19^ In that way, we were able to electrochemically detect single SiNP/enzyme hybrid activity *in situ* at the single particle level. The method might be used for a wide range of applications such as detection of single nano/micro motor activity,^40,41^ monitoring of drug release in real time on a single entity level,^42^ and as the basis for sensing various (bio)catalytic reactions.^5,43,44^
