# Supplementary material for: Catalytic activity of catalase–silica nanoparticle hybrids: from ensemble to individual entity activity
Source: Chem Sci. 2016 Dec 15;8(3):2303–8. doi: 10.1039/c6sc04921d (PMC5363393; doi:10.1039/c6sc04921d)
Supplement: Supplementary file 1 [file SC-008-C6SC04921D-s001.pdf]

Electronic Supplementary Material (ESI) for Chemical Science.
This journal is © The Royal Society of Chemistry 2016

Supporting information

Catalytic Activity of Catalase-Silica Nanoparticle Hybrids: From Ensemble to Individual Entity Activity

Crystal Chan1[a], Dr. Lior Sepunaru1[a], Stanislav V. Sokolov[a],, Dr. Enno Kätelhön[a], Dr. Neil P. Young[b], Prof. Dr. Richard G. Compton*[a]

Materials

Bovine Catalase, Sodium phosphate dibasic, citric acid and sodium chloride (≥99.0%, AR grade) were purchase from sigma. Hydrogen peroxide (~30%) was purchase from Fisher Chemical. The stock concentration was 8.4 M as determined by UV-Vis at 240 nm. Silica nanoparticles (SiNP, r = 59.2 ± 2.8, 5.6 × 10^{12} particles / mL) were purchased from NanoComposix (CA, USA).

SiNP preparation and modification

For all measurements (UV-Vis and electrochemistry), the unmodified SiNPs were used as received and diluted to the desired concentration with ultrapure water from Millipore featuring a resistivity of not less than 18.2 MΩ.cm. The following procedure was used for modifying the SiNP with catalase: 79.5 μL of SiNPs were added to 119.7 μL of water. Next, 20 μL of bovine catalase from stock solution (Sigma, 45 mg/ml) were added. Last, 54.8 μL of 50 mM citrate phosphate buffer (pH=5.4) were added as well. The solution was left overnight for incubation at room temperature. Next, the solution was centrifuged (9000 RPM, Eppendorf 5430-R) for 30 min, the supernatant was removed and the pellet was washed with 219 μL of water and 55 μL of the citrate buffer. The centrifugation and washing process was repeated three times to insure that there is no residual catalase left in solution.

UV-Vis spectroscopy

UV-vis spectroscopy experiments were conducted in citrate-phosphate buffer solution (pH=5.4) using a Shimadzu spectrometer UV- 1800 and quartz cells with a 1 cm optical path. The spectrum of the catalase free in solution (fig. 1c dashed line) was obtained by measuring the absorption of the solution referenced against an identical buffered solution without catalase. The spectrum of the catalase bound to SiNP (fig. 1c solid line) was obtained by measuring the absorption of the solution referenced against an identical buffered solution with similar SiNP concentration without catalase. In order to extract the kinetic data of the SiNP hybrid solution, the absorption at 240 nm was measured as a function of time. In the kinetics measurements, the concentration of the SiNPs was fixed to 0.3 pM with different hydrogen peroxide

concentrations in solution (5 -50 mM). The sample cell was referenced against the same solution containing unmodified SiNP.

TEM

Silica dioxide nanoparticle characterization was performed using a transmission electron microscope (TEM) JEOL JEM-3000F equipped with an EDX spectrometer with an accelerating voltage of 300 kV. Sample preparation involved drop casting nanoparticle suspensions on holey carbon grids (Agar Scientific) and allowing the samples to dry. A size distribution histogram was plotted from the TEM image analysis of 233 NP (Fig. 1b), using ImageJ software. The mean size and standard deviation of the nanoparticles was estimated using a Gaussian fit (Origin 2015).

NTA

A NanoSight LM10 (NanoSight Limited, Amesbury, UK) was used to carry out nanoparticle tracking analysis. A 500 μl sample of SiNP was syringed into the viewing unit of the NanoSight and a red (638 nm) laser was used to illuminate the particles so they could be tracked. Measurements were recorded at 20 0C. NanoSight's NTA software was used to analyse the size distribution and concentration of the NPs.

Electrochemical procedure: electrode preparation and modification

For cyclic voltammetry measurements, a μAutolab type III was used together with a macro glassy carbon electrode (diameter = 3 mm), an SCE (0.241 V vs Standard Hydrogen Electrode) and Pt wire serving as a reference and a counter electrode, respectively. Before all experiments and between experimental repeats the electrode was polished using a diamond spray (Kemet, Kent, UK) in the size sequence of 3.0 μm, 1.0 μm and 0.1 μm to a mirror-like finish, followed by a 2 min sonication to ensure clean and reproducible surface before subsequent functionalization. Experiments were conducted at 25 ^{0}C within a Faraday cage. For all voltammograms reported GPES software was used with a scan rate of 50 mV s^{-1} and in a solution containing 10 mM citrate buffer (pH 5.4) and 20 mM sodium chloride. For the drop cast experimetns, 4 μL of SiNP solution was drop-cast on the electrode surface. The droplet was dried for 20 min under a constant nitrogen flow. The electrochemical measurements were performed immediately after the insertion of the modified electrode into the solution. For all experiments unless stated otherwise, oxygen free solution was prepared by degassing N_2 for at least 15 min prior to measurement (N_2, BOC, Guildford, UK). Oxygen saturated solution was prepared by degassing oxygen for at least 15 min prior to measurement (O_2, BOC, Guildford, UK). For the chronoamperometric measurements a homemade potentiostat was used together with a carbon microelectrode as a working electrode (r = 3.5 μm). Before all experiments the

electrode was polished using micropolish alumina (Buehler) in the size sequence of 3.0 µm, 1.0 µm and 0.1 µm to a mirror-like finish. Data was recorded with a 4 kHz preamplifier filtered with a built-in passive 100 Hz filter. The properties of the homemade potentiostat were described previously.[1]

Impact spikes were analysed using SignalCounter software developed by Dario Omanovic (Centre for Marine and Environmental Research, Ruder Boskovic Institute, Croatia).[2]

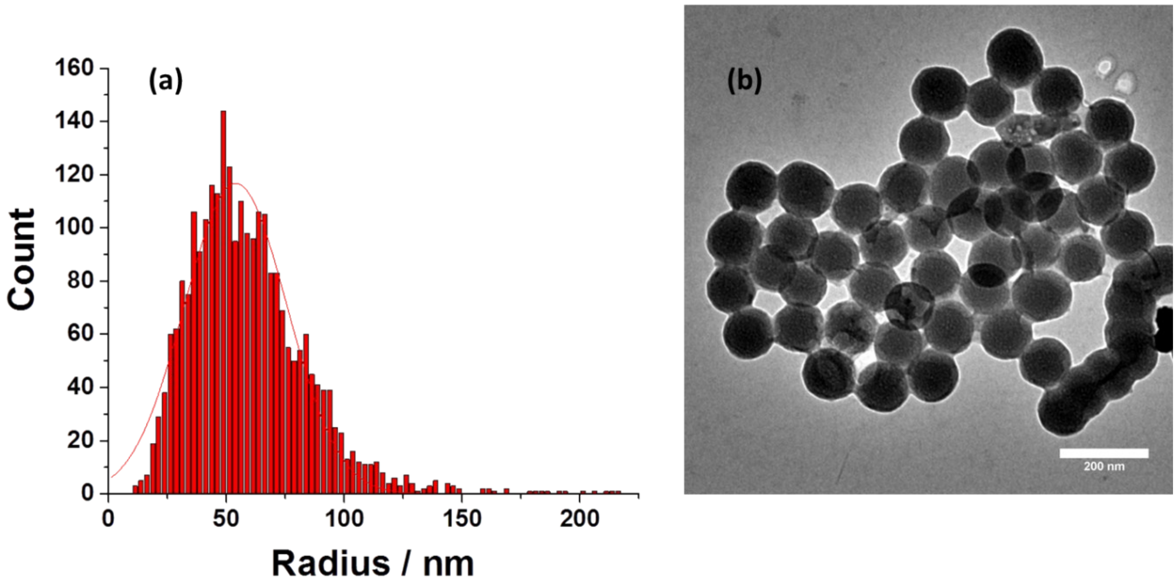

(c)

Particle Type	Measurement 1 (mV)	Measurement 2 (mV)	Measurement 3 (mV)	Average (mV)	Error (mV)
Uncapped SiO2	-28.8	-27.6	-28.4	-28.266	0.6
SiO2@ Enzyme	-3.11	-1.95	-1.86	-2.306	0.625

Fig. S1 (a) NTA of the SiNP-Catalase (b) TEM of bare SiNP and (c) zeta potential of bare and catalase modified SiNP.

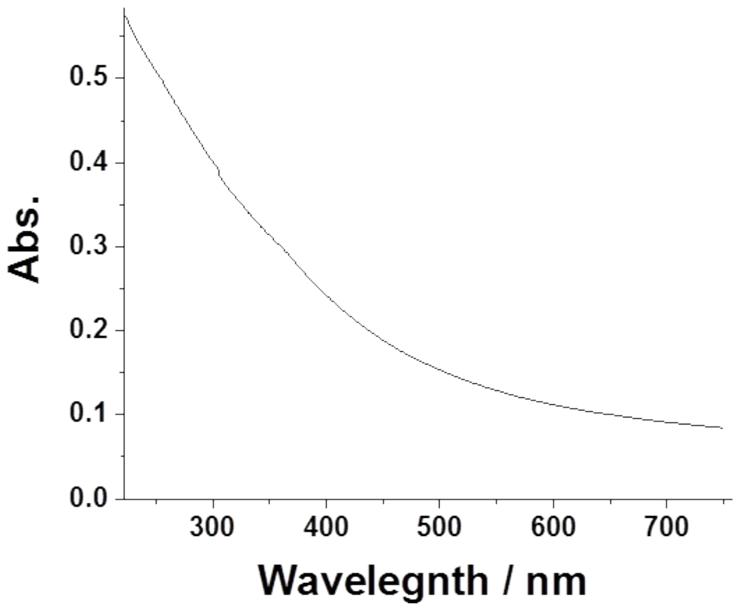

Fig. S2 Absorption spectra of 450 pM SiNP (Unmodified).

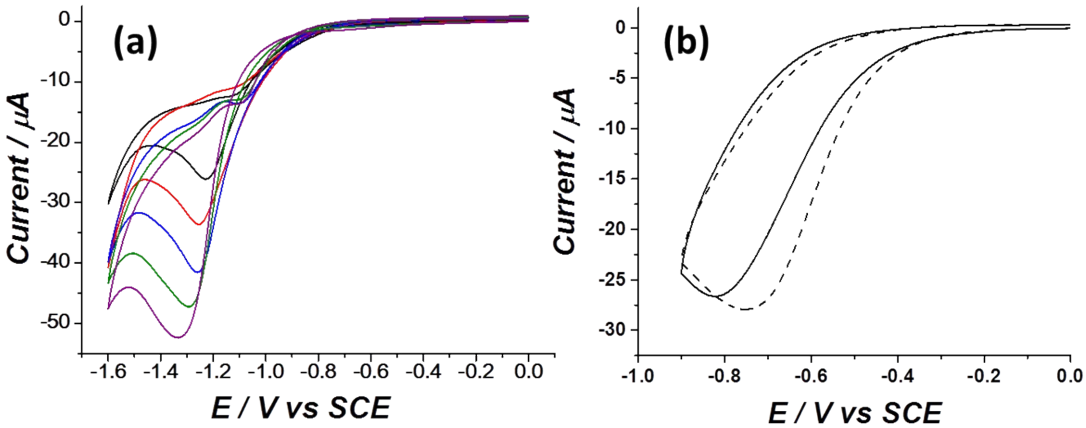

Fig. S3 Voltammetry of (a) 2 mM H_2O_2 solution (υ = 10 – 50 mV s^{-1}) on a bare GCE. (b) O_2 saturated solution on a bare GCE (dashed line) and SiNP modified GCE (solid line).

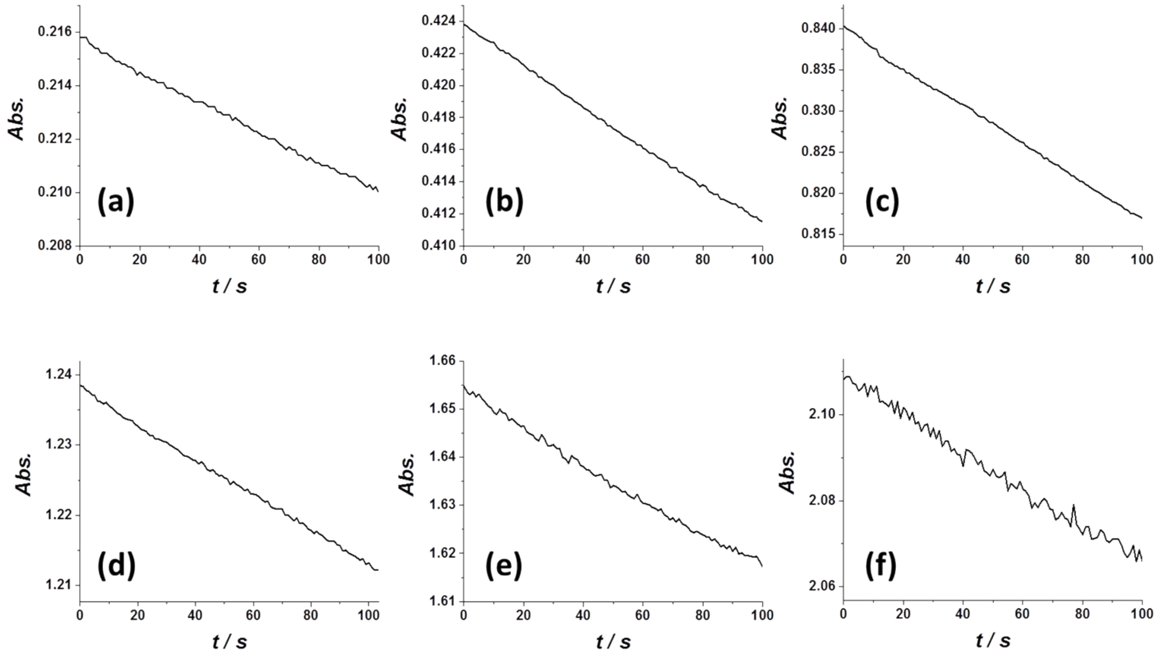

Fig. S4 Absorption at 240 nm as a function of time, for a solution of SiNP/Cat with different H_2O_2 concentrations of (a) 5 mM (b) 10 mM (c) 20 mM (d) 30 mM (e) 40 mM (f) 50 mM.

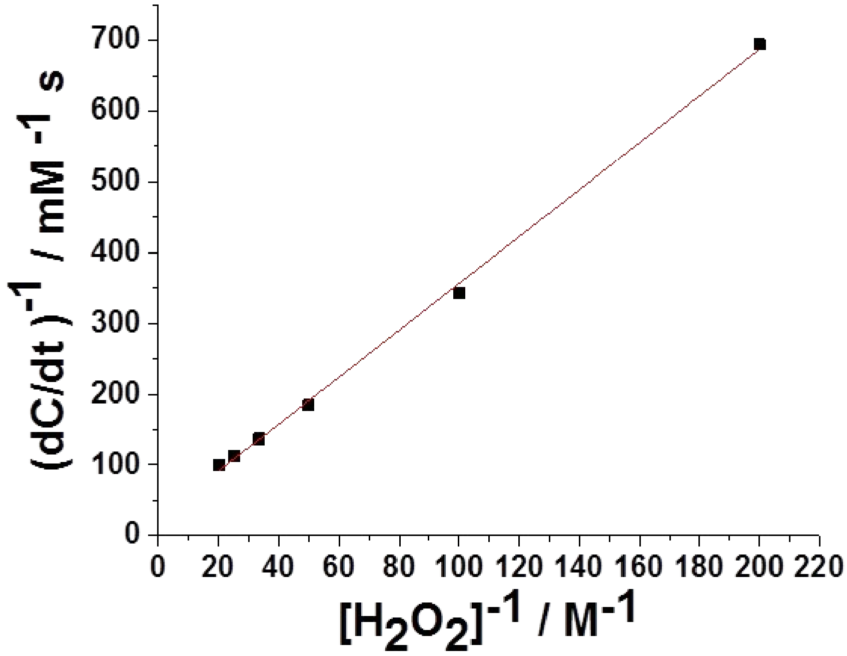

Fig. S5 Lineweaver-Burk plot of the SiNP hybrid activity as a function of hydrogen peroxide concentration (reciprocal plot).

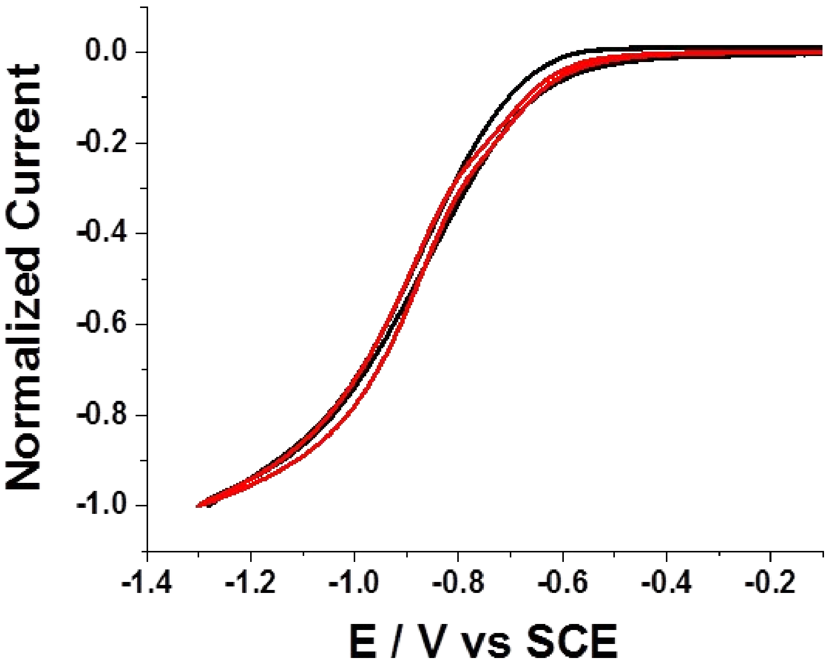

Fig. S6 Normalized currents of the oxygen reduction voltammograms shown in fig. 3b. (black) normalized currents for 'saturated' oxygen solution (red) normalized currents for 'super saturated' oxygen solution. Scan rate was 10 mV s^{-1}, pH =5.4 at 25 ^{0}C.

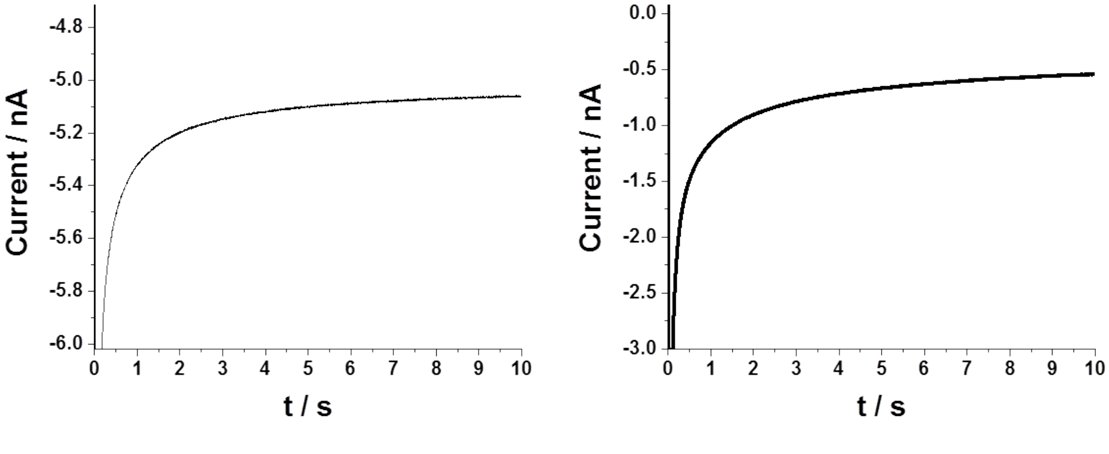

Fig. S7 Chronoamperogram of 20 mM H_2O_2 solution with 100 pM of unmodified SiNP The carbon microelectrode was held at a potential of -1.4 V *vs*. SCE. Measurements were done in citric-phosphate buffer solution (pH =5.4) at 10 mV s^{-1} scan rate at 25 ^{0}C.

Surface coverage of catalase on a SiNP:

The absorption maximum of SiNP/Catalase hybrid ds in solution was at 405 nm and a value of 0.062 was recorded. Using the Beer-Lambert law we can calculate the concentration of bound catalase in solution:

$$A = \varepsilon cl \rightarrow c_{catalase} = \frac{0.063 \pm 0.004}{340000} \approx 185\ \pm 12\, nM$$

Since the concentration of the SiNP in solution was pre-determined to be 0.5 nM, we can estimate the number of catalase enzymes per SiNP to be: $\approx \frac{185\ \pm 12}{0.5} = 370 \pm 24$

The radius of a single SiNP was 59 nm. The radius of catalase is estimated to be 5.12 nm.[3,4] Hence, the maximum number of enzymes that can be loaded on a SiNP can be approximated:

$$\frac{4\pi {R_{SiNP}}^2}{\pi {r_{catalase}}^2} = \frac{4 \times 59^2}{5.1^2} \cong 535\ enzymes/SiNP$$

Our observation of ~ 370 enzymes per SiNP corresponds to 70% monolayer coverage.

Theoretical calculation of SiNP impact frequency:

The steady-state current at a microdisk electrode of radius *r*, assuming a simple *n* electron reduction, is given by

$$I_{SS} = 4nFDCf(\tau)$$

where *n* is the number of electrons transferred, F is the Faraday constant (C mol^{-1}), *C* is bulk concentration (mol cm^{-3}), *D* is diffusion coefficient ($cm^2\ s^{-1}$) and $f(\tau)$ is a function of time, t (s). A convenient single expression for $f(\tau)$ has been obtained from simulation by Shoup and Szabo and shown to correctly predict the current over the entire time domain with a maximum error of less than 0.6%. The Shoup and Szabo expression is: [5]

$$f(\tau) = 0.7854 + 0.8863\tau^{-1/2} + 0.2146\exp(0.7823\tau^{-1/2})$$

where $\tau = 4Dt/r^2$. Multiplication of this by the Avogadro constant, N_A, converts the equation to a form referring to the number of particles. To determine the number of particle impacts expected within a given time, the Shoup–Szabo equation needs to be integrated and this has previously been performed by series expansion.[6] For a 100 pM particles in solution with a radius of 59 nm, the estimated upper value for the average impact frequency is ~ **50 impacts / 10 sec**. The theoretical value is about an order of magnitude higher than the experimentally

observed impact frequency and can be explained by an irreversible absorption process of the NP hybrids to the insulating glass surrounding the active microelectrode.[7]

Theoretical calculation of irreversible two electron reduction of H_2O_2:

The relation of the peak current (Ip) with the scan rate (υ) can be expected to follow the Randles-Ševčík equation for a two electron fully irreversible process:

$$|I_p| = 0.496nFA\left[C_{H_2O_2}\right]\alpha^{\frac{1}{2}}\sqrt{\frac{FD_{H_2O_2}v}{RT}}$$

where I_p is the peak current, α=0.3 is the electron transfer coefficient of the rate determining step, n=2 is the number of electrons transferred and assuming 1st electron transfer is not the rate limiting step, F is the Faraday constant, D_{H2O2} is the diffusion coefficient and equals to $1.71 \times 10^{-9}\ m^2\ s^{-1}$ for hydrogen peroxide.[8] A is the area of the electrode (r=1.5 mm), [$C_{H_2O_2}$] is the hydrogen peroxide concentration, T is the absolute temperature, R is the gas constant and ν is the scan rate.

References

[1] C. Batchelor-McAuley, J. Ellison, K. Tschulik, P. L. Hurst, R. Boldt, R. G. Compton, *The Analyst* **2015**, *140*, 5048–5054.
[2] E. J. E. Stuart, K. Tschulik, D. Omanović, J. T. Cullen, K. Jurkschat, A. Crossley, R. G. Compton, *Nanotechnology* **2013**, *24*, 444002.
[3] M. le Maire, B. Arnou, C. Olesen, D. Georgin, C. Ebel, J. V. Møller, *Nat. Protoc.* **2008**, *3*, 1782–1795.
[4] H. Sund, K. Weber, E. Mölbert, *Eur. J. Biochem.* **1967**, 4, 400–410.
[5] D. Shoup, A. Szabo, *J. Electroanal. Chem. Interfacial Electrochem.* **1982**, *140*, 237–245.
[6] E. J. E. Stuart, Y.-G. Zhou, N. V. Rees, R. G. Compton, *RSC Adv.* **2012**, *2*, 6879–6884.
[7] S. Eloul, R. G. Compton, *ChemElectroChem* **2014**, *1*, 917–924.
[8] D. M. H. Kern, *J. Am. Chem. Soc.* **1954**, *76*, 4208–4214.
